# Trajectories of school refusal: sequence analysis using retrospective parent reports

**DOI:** 10.1007/s00787-024-02419-5

**Published:** 2024-04-11

**Authors:** Laelia Benoit, Edith Chan Sock Peng, Julien Flouriot, Madeline DiGiovanni, Nicolas Bonifas, Alexandra Rouquette, Andrés Martin, Bruno Falissard

**Affiliations:** 1 Université Paris-Saclay, UVSQ, Inserm U1018, CESP, 94807 Villejuif, France; 2 Yale School of Medicine, New Haven, CT USA; 3 APHP-Cochin Hospital, Maison de Solenn, Paris, France; 4 QUALab, Qualitative and Mixed-Methods Lab, a Collaboration Between the Yale Child Study Center and Inserm U1018, New Haven, CT USA; 5 Ecole Normale Supérieure de Lyon, Lyon, France; 6 Service d’Epidémiologie et de Santé Publique, APHP-Paris-Saclay, Le Kremlin-Bicêtre, France

**Keywords:** School refusal, Outcome, Course, Sequence analysis, School attendance problems, Trajectories

## Abstract

School refusal (SR) is a form of school attendance problem (SAP) that requires specific mental health care. Despite improvements in the definition of SAPs, the course of SR is not well characterized. To explore three-year patterns of SR course in children, as reported by their parents, we deployed an anonymous web-based survey. We defined SR onset as the absence of ≥ 2 school weeks during one academic year, combined with emotional distress. We defined standard SR trajectories using sequence analysis of parents’ recollection of three consecutive years of school attendance. We obtained 1970 responses, 1328 (67%) completed by a parent and meeting the definition of SR. Of these, 729 (55%) responses included three years of school attendance recollection. We identified five prototypical trajectories of SR: two profiles for children: *beaded absences* (n = 272), and *rapid recovery* (n = 132); and three for adolescents: *prolonged recovery* (n = 93), *gradual decline* (n = 89), and *rapid decline* (n = 143). We found five distinct trajectories of retrospective recall of SR course. Through pattern recognition, this typology could help with timely identification of SR and implementation of evidence-based interventions to optimize outcomes. Prospective replication of these findings and their field application is warranted.

**Supplementary Information:**

The online version contains supplementary material available at 10.1007/s00787-024-02419-5.

## Introduction

School attendance problems (SAPs) are associated with increased risk of early and permanent school dropout and of developing psychiatric disorders [1–3]. In the longer term, SAPs are linked to economic and social precariousness [1, 4, 5]. Mounting concern about such consequences has led researchers to better define SAPs in an effort to provide early interventions for affected children [1, 6, 7]. Most authors use Kearney’s criteria for problematic absenteeism to identify children with SAPs, namely: (1) missing at least 25 percent of total school time for at least two weeks; (2) experiencing severe difficulty attending classes for at least two weeks with significant interference in a child’s or family’s daily routines; and/or (3) being absent for at least 10 days of school during any 15-week period while school is in session [8]. Heyne et al. [9] differentiated four distinct categories of SAPs: (1) school refusal (SR, compounded by emotional distress); (2) truancy (when the child conceals absenteeism from their parents); (3) school withdrawal (through parental neglect); and (4) school exclusion (through a school decision). In their detailed definition, SR occurs when a young person: (1) is reluctant or refuses to attend school, in conjunction with emotional distress that is usually made manifest by absences, although not necessarily so, as in the case of repeat late arrivals; (2) does not try to hide absences from their parents; (3) does not display antisocial behavior beyond resistance to parental attempts to get them to school; and (4) parents have made reasonable efforts to secure attendance at school [9].

The prevalence of SR ranges between 1 and 5%, with no difference across genders [10, 11]. In the DSM-5, SR is often co-occurring, a clinical presentation sometimes associated with social or specific phobias, or with generalized anxiety, major depressive, oppositional defiant, post-traumatic stress, or adjustment disorders, among others [12]. Three developmental stages have been identified as conducive to SR: between 5 and 6, 10 and 11, [13] or 13 and 15 years of age [14, 15]. SR onset can be gradual or sudden, and return to school can be final or with relapses [16]. Prolonged absence can compromise learning, socialization, and self-esteem, and thus affect later academic and developmental milestones [5, 11]. Among students with SR, co-occurring social phobia and learning difficulties have been found to predict worse functional outcomes than in unaffected peers [17].

Based on a national health survey in France, we conducted a retrospective study based on parental responses. We sought to identify distinctive course and outcome in SR over the first three years of onset.

## Methods

### Design

In partnership with the educational policy department of the French Ministry of Education (DGESCO) and with parent representatives from the School Refusal Association (APS), we developed a web-based survey using Lime Survey software [18]. APS is a parent-led support group that aims to develop better understanding, recognition, and care of SR. The study was approved by the Ethics Evaluation Committee of the National Institute of Health and Medical Research (Inserm, IRB #00003888).

### Instruments

The survey was intended for parents who believe their child suffers or has suffered SR, whether formally diagnosed or not (see survey content in Appendix 1). The survey defined SR upfront: “School refusal is reluctance or refusal to attend school, associated with emotional distress”. The survey asked 37 questions about the index case: (1) socio-demographic characteristics; (2) SR trajectory (age of onset, course); (3) SR clinical characteristics (symptoms, associated diagnoses) (4) clinical care and (5) school flexibility. We did not collect any personally identifying information.

SR course after onset was assessed from retrospective recollection of school attendance and emotional distress from ages 5 to 20 years old. Question #15 began with the stem “This year my child was:” and provided eight forced-answer choices: Present all year and comfortable at school,’ “Present all year but felt poorly at school,” “Absent for less than two weeks,” “Absent between two and four weeks,” “Absent more than a month but less than one trimester,” “Absent one trimester,” “Absent two trimesters,” or “Absent throughout the school year.” Our inclusion criterion was ≥ 2 school week absences for at least one year [9]. The typical school year in France comprises 36 weeks of teaching and 16 of holidays. We defined SR onset as the child’s age at the time of their first school absence lasting at least two weeks.

### Data collection

Between May and October 2018, a link to the anonymous survey was deployed by: (1) DGESCO, through the superintendents of six major educational regions; and (2) APS, through its private Facebook page, followed by 4542 individuals by the end of the data collection interval. The investigators did not have access to email addresses, the private Facebook group, or any personally identifying data.

### Statistical analysis

We compared continuous and categorical variables using ANOVA and Fisher’s Exact Test, respectively. We calculated two-sided p-values with a significance threshold of < 0.05. To refine comparisons between clusters for variables on which the Fisher Exact Test showed that at least one cluster is significantly different from the others (p < 0.05), we tried all possible groupings of clusters in two macro-groups and looked for statistically significant differences (see Appendix 2). In our report of p values, we adjusted for multiple comparisons using the Bonferroni correction. We analyzed descriptive variables using R (Version 3.6.0) [19].

To determine school refusal trajectories, we used sequence analysis, which aims to identify similar series of successive states (“standard sequences”) within a corpus of sequences [20].

We formed clusters using sequence analysis on the variable “This year my child was” for the 3 years after the beginning of SR (including the year of onset), for the subsample of children for whom school attendance data were available for a minimum of three years after SR onset. Each trajectory was considered a unique sequence within a finite number of possibilities, i.e. the eight forced-choice responses. We calculated sequence similarity using the optimal matching algorithm of the TraMineR package (version 2.0–11.1) [20]. We calculated a substitution matrix by converting the eight absenteeism modalities to their equivalent in days and then calculating the difference in days when moving from one state to another. We used the Hubert C index (HC) from the R Weighted Cluster library (version 1.4) to assess the quality of the clusters we obtained. HC compares the partition obtained to the theoretically best partition possible for a given number of groups. The closer the HC is to 0, the more adequate the clusters [21]. We then described the resulting clusters using variables from the following categories: (1) socio-demographics; (2) SR characteristics; (3) clinical care; and (4) school flexibility.

## Results

### Sample

The survey link was opened on 2511 instances, yielding 1970 complete responses, 1413 (73%) of them meeting the definition of SR. We excluded 85 (6%) responses completed by adult subjects who had suffered from SR in their childhood. The analysis included 1328 parental responses, with 1274 (96%) by mothers. We assessed the relevance of our definition of SR: among the 1328 responses included, 99% children displayed at least one criterion that distinguishes SR from other SAPs (i.e., anxiety, panic attacks). Genders were equally distributed (49% female). At the time of questionnaire completion, mean subject age was 14.7 ± 3.2 years, 887 subjects (67%) had partnered or married parents, and 907 (69%) lived with both parents at the time of SAP onset. Parents’ education level was higher than the national average: 679 (51%) had a diploma equivalent or more than 2 years of post-graduate study, compared to 22% in the general French population, ages 25–64 [22]. Most families (n = 1231, 93%) only spoke French at home.

Mean age of SR onset was 13 ± 4 years. During the two years preceding onset, 323 children (24%) had experienced serious illness or the death of a loved one, 311 (23%) a change of school, 213 (16%) a move, and 157 (12%) a change in family organization. Additionally, 823 (62%) experienced some form of pressure from school, their parents, and/or themselves, 634 (48%) been victims of bullying, insults, or threats, and 206 (16%) of physical violence. Prodromal presentations such as oppositional, depressive, anxious, or somatic symptoms were first evident upon or in anticipation of attending school, at a mean age of 8 ± 4 years. 730 (56%) children had taken an IQ test, and 575 (44%) were gifted children (IQ ≥ 130). Risky screen use was reported by 691 participants (52%). SR was associated with at least one other diagnosis among 1,167 children (88%). The most frequent conditions were depression (n = 425, 33%) and social phobia (n = 367, 28%). See Table 1 for further details.

**Table 1:** School refusal characteristics in the survey sample (n = 1328)

N = 1328	Yes
SR definition: > 2 weeks of school absenteeism with emotional distress	1328 (100)
≥ 1 distinctive criterion of SR: diagnosis of SR by a health care provider; panic attacks; anxiety; stomachaches; headaches; sleep problems	1309 (99)
School refusal characteristics	
Diagnosis of SR by a health care provider	930 (70)
Manifestations related to school attendance	
Panic attacks	1,125 (85)
Sleep problems	912 (68)
Stomachaches	1079 (81)
Headaches	811 (61)
Anxiety	837 (63)
Anger	338 (26)
Parents’ perception of risky use of screens (smartphone, video games, social networks)	691 (52)
Performance anxiety	671 (51)
High IQ (Gifted Child)	575 (44)
Separation anxiety	503 (38)
Complaints toward:	
School	459 (35)
Classmates	338 (26)
Teachers	302 (23)
Drop in academic performance	399 (30)
Arguments with authority figures	388 (29)
≥ 1 associated condition diagnosed by a care provider	1167 (88)
Depression	425 (33)
Social Phobia	367 (28)
Learning disability (e.g., dyslexia, dyspraxia, dysgraphia, dyscalculia)	269 (20)
Attention-Deficit/Hyperactivity Disorder (ADHD)	142 (11)
Post-traumatic stress disorder	100 (8)
Aggravation of preexisting medical conditions (e.g., asthma, diabetes)	87 (7)
Eating disorder (i.e., anorexia, bulimia)	85 (7)
Generalized anxiety disorder	71 (5)
Autism spectrum disorder	67 (5)
Bipolar disorder	12 (1)
Obsessive compulsive disorder	17 (1)
Schizophrenia	6 (0.5)
Relationship with professionals	
Sense of understanding: kindness and availability of at least some interlocutors	
School	908 (68)
Healthcare providers	1040 (78)
Sense of rejection: criticism, threat, judgments	
School	354 (27)
Healthcare providers	103 (8)

Values represent n (%), unless noted otherwise

Regarding school adaptations, 759 children (61%) had adjusted school schedules (such as reduced timetables or personal support plans), and 354 parents (27%) felt rejected or criticized by school professionals. Half of the children (n = 681, 54%) received at least one pharmacological treatment, and for 546 (44%), the first health professional consulted was their family doctor. Half of the families spent more than 150 euros/month in non-reimbursed care (min ≤ med ≤ max: 0 ≤ 150 ≤ 3000), and 912 (69%) parents adjusted their work schedules to adapt to their child’s SR.

### Trajectories of school refusal during the first three years after onset

Retrospective recall of school attendance during the first three years after SR onset was provided for 729 (55%) subjects. Individual trajectories of SR during this time are represented in Supplementary Fig. 1 using *dendrograms*, tree diagrams depicting taxonomic relationships. Absences of less than two weeks or between two weeks and one month were observed among 211 (29%) and 175 (24%) children during the second and third year after SR onset, respectively. Complete return to school and return to school with discomfort were observed among 124 (17%) and 146 (20%) children during the second and third years, respectively.

**Fig. 1:**
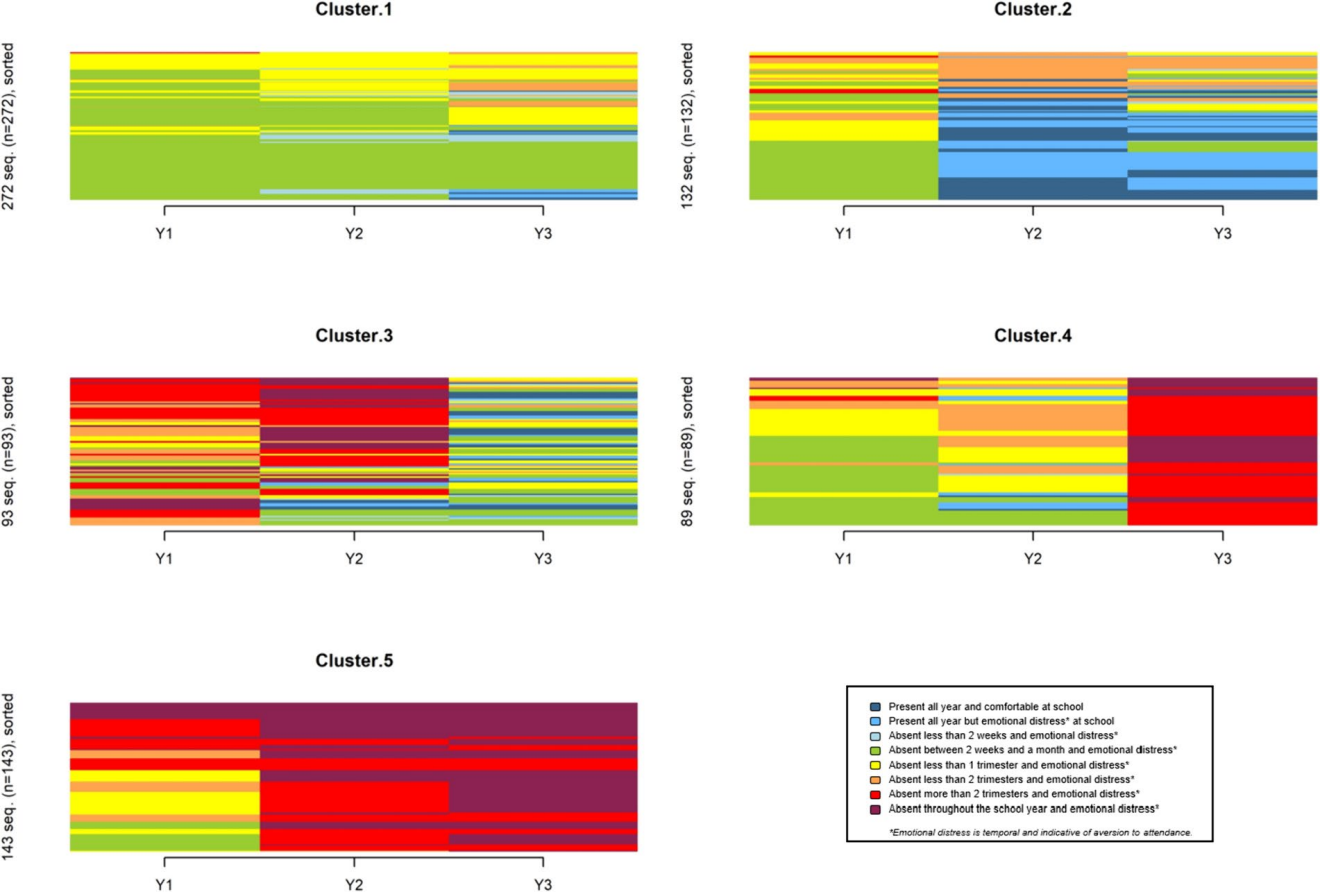
Five trajectories of school refusal during the first three years after onset

We set the optimal number of clusters at five, following the optimal matching algorithm (Supplementary Fig. 2). An HC of 0.1 confirmed the robustness of fit for these five clusters. Children’s individual trajectories of SR according to each cluster are depicted in Fig. 1.

### Characteristics of five SR trajectory clusters

Table 2 shows socio-demographic characteristics, SR characteristics, associated diagnoses, characteristics of care and school flexibility across the five clusters. Age of onset was lower in Clusters 1 and 2, “children,” than in clusters 3, 4, and 5 “adolescents” (p < 0.001).

**Table 2:** Sample characteristics, clinical care, and school flexibility across five school refusal trajectories (n = 729)

Cluster description (n)	1 Beaded absences (272)	2 Rapid recovery (132)	3 Prolonged recovery (93)	4 Gradual decline (89)	5 Rapid decline (143)	Total sample (729)	Cluster comparisons	p-value	Significance	Odd ratio
Sociodemographic characteristics										
Sex: male	140 (52)	73 (55)	40 (43)	43 (48)	72 (50)	368 (50)	**–**	**–**	**–**	**–**
School refusal characteristics										
Age at onset (> 2 weeks of absence): mean years (SD)	9 (3)	10 (3)	12 (3)	11 (3)	12 (2)	11 (3)	{3,4,5} > {1,2}	< 0.001	***	
Complaints toward School	114 (42)	47 (36)	25 (27)	31 (35)	36 (25)	253 (35)	{1,2,4} > {3,5}	< 0.001	***	1.83
Complaints toward Teachers	77 (28)	38 (29)	13 (14)	23 (26)	19 (13)	170 (23)	{1,2,4} > {3,5}	< 0.001	***	2.48
Complaints toward Classmates	83 (30)	42 (32)	14 (15)	27 (30)	21 (15)	187 (26)	{1,2,4} > {3,5}	< 0.001	***	2.56
Anger about going to school	72 (27)	27 (21)	22 (24)	36 (40)	30 (21)	187 (26)	{4} > {1,2,3,5}	0.001	***	2.2
Opposition: Refusal to attend appointments or hostility toward authority figures	130 (48)	54 (41)	30 (32)	52 (58)	68 (48)	529 (46)	{1,2,4,5} > {3}	0.003	**	1.92
							{4} > {1,2,3,5}	0.008	**	1.78
Headaches	189 (70)	76 (58)	52 (56)	54 (61)	82 (57)	453 (62)	{1} > {2,3,4,5}	0.001	***	1.66
Stomachaches	248 (91)	102 (77)	75 (80)	74 (83)	115 (80)	614 (84)	{1} > {2,3,4,5}	< 0.001	***	2.57
Gender identity issues	3 (1)	4 (3)	6 (6)	1 (1)	7 (5)	21 (3)	{2,3,5} > {1,4}	0.004	**	4.32
Associated diagnoses										
Aggravation of preexisting medical conditions (e.g., asthma, diabetes)	22 (8)	23 (17)	7 (8)	7 (7)	5 (4)	64 (9)	{1,2,3,4} > {5}	0.006	**	3.09
							{2} > {1,3,4,5}	< 0.001	***	2.86
Learning disability (e.g., dyslexia, dyspraxia, dysgraphia, dyscalculia)	73 (27)	38 (29)	19 (21)	12 (13)	26 (18)	174 (23)	{1,2,3} > {4,5}	0.002	**	1.81
							{1,2} > {3,4,5}	0.001	***	1.78
Social phobia	69 (25)	37 (28)	35 (38)	32 (36)	63 (44)	236 (32)	{5} > {1,2,3,4}	0.001	***	1.88
							{3,4,5} > {1,2}	< 0.001	***	1.87
Clinical care										
SR diagnosis by a health care provider	186 (68)	91 (69)	81 (87)	67 (75)	118 (83)	543 (74)	{3} > {1,2,4,5}	0.001	**	2.54
							{3,5} > {1,2,4}	< 0.001	***	2.33
Follow-up by a psychiatrist/child and adolescent psychiatrist	171 (63)	81 (61)	72 (77)	70 (78)	112 (78)	506 (69)	{3,4,5} > {1,2}	< 0.001	***	2.16
Renounced some care for financial or organizational reasons (ns)	159 (58)	72 (55)	41 (44)	46 (51)	76 (53)	394 (54)	–	–	–	–
Frequency of care appointments: at least once a week*	70 (26)	41 (31)	33 (35)	18 (20)	44 (31)	206 (28)	ns	ns	ns	ns
Psychotherapy (> 1 type): individual; family; group therapy	62 (23)	40 (30)	27 (30)	28 (31)	63 (44)	220 (30)	{5} > {1,2,3,4}	< 0.001	***	2.15
Inpatient hospitalization (in psychiatry or pediatrics)	58 (21)	30 (23)	26 (28)	25 (28)	54 (38)	193 (26)	{5} > {1,2,3,4}	0.001	***	1.95
Psychotropic medication										
Antidepressant	84 (31)	39 (30)	49 (53)	42 (47)	63 (44)	277 (38)	{3,4,5} > {1,2}	< 0.001	***	2.06
Antipsychotic	11 (4)	3 (2)	3 (3)	5 (6)	15 (10)	37 (5)	{5} > {1,2,3,4}	0.002	**	3.0
Perceived as efficient	45 (17)	19 (14)	24 (26)	12 (14)	14 (10)	114 (16)	{3} > {1,2,4,5}	0.005	**	2.11
> 1 type: antidepressant; anxiolytic; sleeping pill; ADHD medication; antipsychotic; herbal anxiolytic	71 (26)	26 (20)	36 (39)	35 (39)	47 (32)	215 (29)	{1,3,4,5} > {2}	0.004	**	1.89
							{3,4,5} > {1,2}	< 0.001	***	1.8
Judicial involvement or protective service report	28 (10)	19 (14)	8 (9)	17 (19)	31 (22)	103 (14)	{4,5} > {1,2,3}	0.001	***	2.1
Separation of children from parents suggested	52 (19)	22 (17)	27 (29)	25 (28)	55 (38)	181 (25)	{5} > {1,2,3,4}	< 0.001	***	2.28
							{3,4,5} > {1,2}	< 0.001	***	2.19
School flexibility										
School accommodation (reduced timetable, personal support plan)	88 (32)	59 (45)	47 (51)	41 (46)	56 (39)	291 (40)	{2,3,4,5} > {1}	0.001	***	1.67
							{2,3,4} > {1,5}	0.001	***	1.66
Change of school	106 (39)	62 (47)	54 (58)	45 (51)	74 (52)	341 (47)	{2,3,4,5} > {1}	0.001	***	1.66
Home schooling	42 (15)	28 (21)	19 (20)	32 (36)	55 (38)	176 (24)	{4,5} > {1,2,3}	< 0.001	***	2.75
							{5} > {1,2,3,4}	< 0.001	***	2.4
Meeting with the education officer	150 (55)	57 (43)	52 (56)	58 (65)	78 (55)	736 (54)	{1,3,4,5} > {2}	0.003	**	1.72
Relationship with school: Sense of rejection	74 (28)	54 (41)	28 (30)	27 (30)	43 (30)	254 (31)	{2} > {1,3,4,5}	0.005	**	1.71

Values represent n (%), unless noted otherwise

*p < 0.05, **p < 0.01, ***p ≤ 0.001. For cluster comparisons, we reported only results with the lowest p-value and sorted them out from the highest odd-ratio to the lowest. We reported all cluster comparisons in Appendix 2

### Clusters 1 and 2: young children

The average age of onset for children in clusters 1 and 2 was respectively 9 ± 3 years and 10 ± 3 years, the youngest clusters in this study (p < 0.001). These children were more likely to have a diagnosis of learning disabilities (OR = 1.78, p < 0.001) than participants in clusters 3, 4 and 5. They were less likely to receive follow-up by a psychiatrist or a child psychiatrist (p < 0.001), antidepressants (p < 0.001) and multiple psychotropic medications (p < 0.001), than children in clusters 3, 4 and 5.

#### Cluster 1: beaded absences

This cluster, the largest (n = 272, representing 37% of all respondents), describes a moderate, stable course over the first three years, with young children presenting with only short-term absences. Almost all children in cluster 1 (n = 267, 98%) had at least one somatic symptom. They were more likely to experience stomachaches (n = 289; 91%, p < 0.001, OR = 2.57) and headaches (n = 189; 70%, p = 0.001, OR = 1.66) than the four other clusters. The latter were more likely to benefit from school accommodations (OR = 1.67, p = 0.001) or a change of school (OR = 1.66, p = 0.001), than children in cluster 1.

#### Cluster 2: rapid recovery

This profile encompasses a high proportion of children with existing medical problems who tended to return to school by the second year of follow-up. In this cluster (n = 132), SR was associated with a worsening of preexisting medical conditions more frequently (17%) than in the four other clusters (n = 23, p < 0.001). The three most common preexisting medical conditions were asthma, rare and/or chronic diseases, and digestive problems. Compared to the four other clusters, parents in cluster 2 reported more frequently not having met the education officer (OR = 1.72, p = 0.003) and feeling rejected by the school (n = 54, 41%, OR = 1.71, p = 0.005).

If considered together with cluster 4, participants in cluster 1 and 2 reported higher levels of complaints toward school (OR = 1.83, p < 0.001), teachers (OR = 2.48; p < 0.001), and classmates (OR = 2.56; p < 0.001) than participants in clusters 3 and 5.

### Cluster 3, 4, and 5: adolescents

In clusters 3, 4 and 5, encompassing older children, participants were more likely to receive psychiatric follow-up (OR = 2.16, p < 0.001), a diagnosis of social phobia (OR = 1.87, p < 0.001), antidepressant medication (OR = 2.06, p < 0.001) or multiple medication (OR = 1.8, p < 0.001) than children in clusters 1 and 2. A separation of children from parents was more likely to be suggested for these three clusters (OR = 2.19, p < 0.001) than for participants in clusters 1 and 2.

#### Cluster 3: prolonged recovery

For this cluster, SR onset was on average at age 12 ± 3. Children in the four other clusters were more likely to display oppositional behavior than the ones in cluster 3 (OR = 1.92, p = 0.003). Contrary to clusters 4 and 1, children in clusters 2, 3 and 5 were more likely to question their gender identity, although still infrequently (OR = 4.32, p = 0.004). Participants in cluster 3 obtained a formal SR diagnosis more often than other clusters (87%, n = 81, OR = 2.54; p = 0.001) and were more likely to perceive medication as helpful (n = 24, 26%, OR = 2.11, p = 0.005). These children may have benefited from an intensive healthcare response: 35% of them had at least one care appointment per week (n = 33, p = 0.02, cluster comparison = ns) and they were less likely to forego care for financial or organizational reasons (n = 41, 44%, ns).

#### Cluster 4: gradual decline

SR started on average at 11 ± 3 years for the children in this cluster (n = 89, representing 12% of respondents). They maintained partial school attendance during the first two years, followed by cessation during the third. In comparison to the four other clusters, children in this cluster were more frequently angry about going to school (n = 36, 40%, OR = 2.2, p = 0.001), and more likely to express oppositional behavior (n = 52, 58%, OR = 1.78, p = 0.008). As mentioned above, participants in clusters 1, 2 and 4 complained more frequently about their school, teachers, and classmates than children in clusters 3 and 5.

#### Cluster 5: rapid decline

In this profile, with an age of onset at 12 ± 2 years, school absenteeism persisted despite accommodations and medical monitoring. This cluster was the most likely to report social phobia, 44% (n = 63, OR = 1.88, p = 0.001). Children in this group had an intensive healthcare response. Compared to the four other clusters, participants in this cluster were more likely to receive more than one type of psychotherapy (44%, n = 63, OR = 2.15, p < 0.001), to having been hospitalized in a psychiatry or pediatric unit (38%, n = 54, OR = 1.95, p = 0.001), and to receive antipsychotic prescriptions (10%, n = 15, OR = 3, p = 0.002). This cluster featured the highest frequency of homeschooling, for 38% of participants (n = 55, OR = 2.4, p < 0.001). Among these adolescents, a separation from parents was more likely to be suggested than in the four other clusters (OR = 2.28, p < 0.001).

If considered together, participants in clusters 4 and 5 were more likely to feature homeschooling (OR = 2.75, p < 0.001) and to be subject to judicial or social services report (OR = 2.1, p = 0.001) than children in clusters 1, 2 or 3.

## Discussion

### Latent profiles

By acknowledging the varied factors associated with each subtype of SR, this common clinical presentation can be divided into more comprehensible, discrete patterns for parents, educators, and healthcare providers to identify. Through pattern recognition, such a typology could help with timely identification of SR and implementation of evidence-based interventions to optimize SR outcomes.

Across all profiles, parents reported how their children’s attendance problems were frequently associated with related conditions, ranging from being identified as gifted (potentially feeling out of place at school), to suffering from bullying, depression, social phobia, or gender identity issues. Somatic complaints were also common across profiles, in keeping with existing literature [23, 24]. The high frequency of these concurrent concerns is arguably unique to SR within the broader domain of SAPs, as the definition of SR encompasses the child’s emotional distress [9]. SR may appear less stigmatizing than other SAPs and thus have an element of desirability for parents attempting to make sense of a difficult situation. Previous studies on giftedness have shown that parents look for psychometric testing to provide them and the schools with “evidence” that the distress of their child is real [25]. Of note, the typical margins of error for IQ tests increase as the IQ measure increases, and is estimated at 5 percent for gifted children [26]. Nevertheless, the “long recovery” profile reports rapid medical and school support, echoing existing evidence suggesting that early and intensive care is associated with more favorable outcomes [27].

### Parental involvement and school flexibility

We found that a high degree of parental involvement may be associated with a better outcome, but only for certain interventions. Adaptation of parental work schedule was frequently reported across profiles. Alternatively, home-schooling was significantly higher for the two groups with more negative outcomes (“progressive worsening” and “rapid worsening”). Homeschooling may be a consequence of a prolonged absence, but this association may also be confounded by age: the average age of favorable profiles was lower than of unfavorable profiles, suggesting that the benefit may be a function of earlier intervention. Also, home schooling requires self-discipline and may therefore not be offered to younger children.

To support parents’ efforts, schools serve as a second pillar in supporting favorable outcomes. School accommodations were least frequently reported for younger children, in the “beaded absences” and “rapid recovery” profiles. The “rapid recovery” profile was more likely to be associated with a physical illness or learning disability rather than psychiatric illness, suggesting that the intensity of school intervention may not be as significant for these children as for other profiles, instead favoring treatment of the underlying medical or learning condition. In response to the high frequency of visits to the school nurse, as reported in this study, school-based attendance teams have been recommended to improve detection of risk factors for SR by monitoring students’ emotional distress, somatic symptoms, and parental motivations for absence [28].

### The role of healthcare providers

Healthcare providers are a third key stakeholder for children dealing with SR, and can provide additional screening, treatment, and advocacy for the conditions that contribute to SR. Nearly half of our sample had their first point of contact for SR with their family physician, and the overwhelming majority reported at least one associated diagnosis. Healthcare providers can support parents and schools by tailoring their approach according to SR subtype. For younger patients, SR may be more likely related to physical illness, so diagnosis of a physical illness at a doctor’s visit can include conversations with parents and schools about how to minimize distress and disruptions to school attendance. For older patients, healthcare providers can act as advocates between the school and mental health systems to address bullying, incorporate flexible school schedules, and recommend intensive mental health care. Among healthcare providers, school nurses can play a pivotal role [29].

Regarding specific profiles, psychiatric medication use was more common for the older three profiles than for the younger two, as was follow-up with a psychiatrist, further suggesting a distinction between the younger child/medical or learning comorbidity-associated profiles, and the older child/psychiatric comorbidity-associated profiles. The “long recovery” profile, with high rates of diagnosis by a care provider, frequent appointments, and high satisfaction regarding medication, was associated with an eventual successful return to school after intensive support. Interestingly, psychiatric hospitalization was more frequent for the older profiles, but was not associated with any clear benefit for resolving SR. Qualitative research of psychiatric healthcare for adolescents struggling with SR suggests that adolescents find mental healthcare valuable in assisting with self-transformation and problem-solving abilities [30]. Additionally, a meta-analysis of the existing treatments for SR demonstrates that most interventions focus on cognitive behavioral therapy [31]. Together, these findings may explain the limited association of hospitalization and return to school demonstrated in this study: Acute hospitalizations may be more likely to focus on crisis stabilization rather than on the longer-term psychological coping that appears to be most valued and effective by adolescents.

An additional point of intervention for healthcare workers is to monitor screen time: Risky screen use was reported by most participants across clusters. Increased screen time questions family functioning as a trigger or maintenance factor in SR [32], and therefore screen time monitoring should arguably include an assessment of the family’s ability to be engaged in the child’s trajectory. On the other hand, given that nearly a third of participants reported a feeling of rejection or criticism by the schools as relevant to their child’s trajectory, it is paramount that healthcare providers first build rapport with families to support them in being engaged with their child’s school trajectory, to avoid coming across as judgmental or intrusive. Parents of school-refusing children have already been shown to perceive a lack of understanding from schools in addressing their children, and the same dynamic should be avoided in healthcare [33]. Potential interventions may also focus on parental self-efficacy, a concept shown to be implicated in SR, although not quite predictive once controlling for child or parent comorbidities [34].

### Underrepresented minorities with SR

The vast majority of children in this study only spoke French at home, illustrating a known difficulty in accurately characterizing the SAP of children of underrepresented families (immigrants, ethnic minorities) [35] due to both a lack of representation in parental support groups and a mischaracterization of students’ and parents’ behaviors [36, 37]. Indeed, the definition of parental “reasonable efforts” has been criticized for being left to the interpretation of professionals [37, 38]. Nevertheless, parents from underrepresented groups may experience difficulties in communicating their concerns to school personnel [36], which may lead SR to be categorized as truancy or school withdrawal instead [37]. Similarly, young people from underrepresented groups experiencing emotional distress can feel ashamed to ask for help [39], leading to the conflation of truancy and SR.

### Limitations

We acknowledge several limitations, the main one being the retrospective nature of this study. Faulty and limited recall by parents, memory distortion, and recollection bias, all need to be considered. This study describes associations only, and therefore we cannot make any causative claims about what features of a trajectory are protective or harmful for a given child profile, limiting the strength of recommendations for future practice. The survey we used was newly created, and its development and metrics not fully tested prior to deployment. Sequence analysis is a non-parametric cluster analysis technique, also called non-supervised clustering technique, which requires important sample sizes. Therefore, further confirmatory studies should include a larger sample. Previous studies about school absenteeism [38, 40, 41] used instead an inductive cluster analysis technique, in which different time patterns are first identified, then used as one variable. The combination of a non-representative and reduced analytic sample are shortcomings of our study.

Participants were invited based on self-identification, not by formal delineation of SR versus other SAP, so it is unclear whether children described in this sample truly suffer from SR, as opposed to truancy, school withdrawal, or school exclusion. To more reliably characterize the subtypes of attendance problems, screening instruments such as the School Non-Attendance ChecKlist (SNACK) are being developed and may guide future studies once sufficient validity evidence has been gathered to support scoring and uses [9, 42]. Also, we noted a high frequency of parental involvement in our sample: Parents who are more involved may be more likely to participate in such a study. It is important to note that parental flexibility is a resource that not all children have access to; in our sample most children lived with both parents, who had higher employment and education metrics than average, such that this study does not adequately characterize the population of children without these presumably protective factors. Parents may have different thresholds to evaluate their child as having an addiction. This study may not accurately characterize the SAP of children of minority families due to their underrepresentation in the survey sample. Finally, future studies could explore the SR profiles based not only on parental reporting but on child self-reporting.

## Conclusion

This study on subtypes of SR trajectories based on parental perspectives emphasizes seeking support for the distress central to SR. We hope our findings can help inform tailored supports for each profile of SR from three key stakeholders: parents, schools, and healthcare workers. A sense of trust and non-judgment between families, schools, and other providers is key toward creating an environment for children to remain engaged in their education and to cope with stressors in an adaptive way.

## Supplementary Information

Below is the link to the electronic supplementary material.

Supplementary file1 (DOCX 37 KB)

Supplementary file2 (DOCX 31 KB)

Supplementary file3 (DOCX 729 KB)

## Data Availability

Data is available upon reasonnable request to the corresponding author.
