# Supplementary material for: Trajectories of school refusal: sequence analysis using retrospective parent reports
Source: Eur Child Adolesc Psychiatry. 2024 Apr 11;33(11):3849–59. doi: 10.1007/s00787-024-02419-5 (PMC11588807; doi:10.1007/s00787-024-02419-5)
Supplement: Supplementary file 1 — Supplementary file1 (DOCX 37 KB) [file 787_2024_2419_MOESM1_ESM.docx]

# Appendix 1: Survey content

# Title: Trajectories of school refusal – Better understanding for better care.

# FRONTPAGE

## Who is this survey for?

To address school absenteeism, the French Ministry of Education, the National Institute of Health and Medical Research (Inserm), with parent representatives from the School Refusal Association (*Association Phobie Scolaire*), have developed a scientific assessment of "school refusal" (also called anxious school refusal, or school phobia). Please answer this survey if you think your child experienced school refusal.

## What is school refusal?

School refusal is defined as avoidance or refusal to go to school combined with emotional distress. These students refuse to attend school and display extreme anxiety when forced to do so (e.g., clinging to the school gates, vomiting on the way, and running away). This behavior was previously known as "school phobia" and is sometimes called "anxious school refusal."

Children with school refusal do not try to hide absences from their parents. They do not display oppositional or antisocial behavior beyond resistance to parental attempts to get them to school, and you, as a parent, have made reasonable efforts to secure attendance at school.

If in doubt, you can ask the parent representatives from the Association Phobie Scolaire about your child's situation: <https://www.phobiescolaire.org/>

# SURVEY

You think your child has experienced "school refusal." Thank you for answering this survey. Your experience will provide valuable information to better support young people and their parents at school and in medical settings.

Before answering, you may want to have your child's report cards and health care assessments at hand. They can help you remember important details. This survey takes about 20 minutes. If you must take a break, you can save your answers and resume the survey later.

We designed the survey to collect all information from only one child's parent. We will ask questions about the other parent, but you can complete them yourself. However, if you have more than one child with school refusal, please fill out one survey per child.

This survey is anonymous and has been approved by the Inserm Ethical Evaluation Committee (IRB00003888). Contact: Dr Laelia BENOIT, laelia.benoit@inserm.fr. The results are confidential. If you need help filling out this questionnaire, please ask the professionals at your child's school.

**Question #1: How did you learn about this survey?**

- By your child's school
- By a health professional
- By the Association Phobie Scolaire or its Facebook group
- By a relative (family, friend)
- Through your online social network (Twitter, Facebook...)
- Other

**Question #2: Who are you**

- Mother
- Father
- Other

**Question #3: What is the sex of your child?**

- Female
- Male

**Question #4: What is the year of birth of your child?**

List (one choice): [1980; …; 2012]

**Question #5: What is your relationship with the other parent?**

- Partners or Married
- All other options: separated, divorced, the other parent is deceased, single parent (adoption, solo fertility treatment), etc.

**Question #6: What is your highest level of education?**

- At least two years of undergraduate education *(Examples in French: DUT, BTS, DEUG, License, maîtrise, master, doctorat, etc.)*
- Less than two years of undergraduate education *(Examples in French: Primaire, College, Lycée, CAP, BEP, Terminale).*

**Question #7: What is the other parent's highest level of education?**

- At least two years of undergraduate education *(Examples in French: DUT, BTS, DEUG, License, maîtrise, master, doctorat, etc.)*
- Less than two years of undergraduate education *(Examples in French: Primaire, College, Lycée, CAP, BEP, Terminale).*

**Question #8: Is there another language than French spoken at home?**

- Yes
- No

If Yes: which language? **[short text]**

**Question #9: We want to trace your child's school history.** What grade did your child attend the year they turned [**age**]? Once you have reached your child's current age, please leave the rest of the table blank. Example: Chloe is eight years old. She entered first grade the year she turned 5. She then skipped second grade and repeated third grade.

| **Age (years)** | **Class** |
| --- | --- |
| **5** | First-grade *(In French: CP)* |
| **6** | Third grade *(In French: CE2)* |
| **7** | Third grade *(In French: CE2)* |
| **8** | Fourth grade *(In French: CM1)* |

| **Age (years)** *(from 5 to 20 years old)* | **Class** |
| --- | --- |
| 5 | Short text |
| 6 | Short text |
| … | Short text |
| 20 | Short text |

**Question #10: Before the onset of the school refusal, did your child experience school pressure?**

- Yes, from the school
- Yes, from one or both parents
- Yes, from the child itself (self-pressure)
- No, I don't think so.

**Question #11: Before the onset of school refusal, did one of these events happen?**

- Change of school
- Move
- Change in family structure (i.e., divorce)
- Serious illness or death of a loved one
- No, none of these events happened.

**Question #12: Before the onset of school refusal, has your child suffered violence?**

- Bullying, Insults, or Threats
- Physical violence
- Theft
- Sexual assault
- No, not that I know of.
- Other

**Question #13: Before or during the school refusal, has your child questioned:**

- Their origins (i.e., migration, adoption, etc.)
- Their sexual orientation (i.e., homo/heterosexuality, etc.)
- Their gender identity (i.e., female/male, etc.)
- No, not that I know of
- Other

**Question #14: Before the school refusal, did your child have a chronic illness (i.e., diabetes, asthma, chronic disease, cancer, etc.)?**

- Yes
- No

**SCHOOL REFUSAL ONSET**

**Question #15: We want to assess the course of your child's school absences due to school refusal.** After school refusal onset, returning to school is not straightforward, and relapses are frequent*.

Please select your child's age on the left side of the table, and select the presence or absence of your child on the right side of the table.

*We only assess absences due to emotional distress (fear, anxiety). If your child was absent from school without emotional distress (i.e., travel, illness, opposition towards authority), please select the option "present without emotional distress".

| Age [5 – 20] | School trajectory |
| --- | --- |
| 5 | [list: select one in eight forced choices] |
| 6 | [list: select one in eight forced choices] |
| 7 | [list: select one in eight forced choices] |
| 8 | [list: select one in eight forced choices] |
| … | … |
| 20 | [list: select one in eight forced choices] |

| List of forced choices | |
| --- | --- |
| #1 | Present all school year and feeling comfortable at school |
| #2 | Present all school year but experiencing emotional distress |
| #3 | Absent for less than two weeks (with emotional distress) |
| #4 | Absent between 2 weeks and one month (with emotional distress) |
| #5 | Absent for less than three months (with emotional distress) |
| #6 | Absent for less than six months (with emotional distress) |
| #7 | Absent for more than six months (with emotional distress) |
| #8 | Absent the entire school year (with emotional distress) |

**Question #16: Who thought first that your child might have "school refusal"?**

- A care provider
- A school worker
- Yourself (i.e., online search, etc.)
- Your child
- Your relatives
- Other

**Question #17: Was the diagnosis of school refusal confirmed by a healthcare provider?**

- Yes
- No

**Question #18: How does the school refusal of your child manifest?**

- Complaints about school
- Complaints about teachers
- Complaints about classmates
- Anxiety related to school attendance
- Anger about going to school
- Drop in academic performance
- Social isolation
- Panic attack
- Change in eating habits (i.e. loss of appetite, weight gain)
- Scarifications
- Depressed mood, sadness, guilt
- Suicidal ideations
- Sleep problems: insomnia
- Performance anxiety
- Separation anxiety (the child gets nervous about being without their parents or wants to spend more time with their parents)
- Opposition: refusal to attend appointments, hostility towards authority figures
- Frequent visits to the school nurse
- Headaches
- Stomachaches
- Vomiting
- Diarrhea
- Tachycardia (feeling that the heart is racing)
- Abnormal movements without a cause (paralysis, epileptic-like crises, etc.)
- Dizziness
- Worsening of pre-existing illness (i.e., diabetes, asthma)
- Other

**Question #19: Describe your relationship with the school of your child after the onset of school refusal**

- Feeling of understanding: kindness and availability of at least some school workers
- Feeling of rejection: criticism, judgments, and threats
- Other

**Question #20: Have you met with the following school workers?**

- Education Officer
- Teacher(s)
- School director
- School care provider (nurse, doctor)
- School social worker
- I haven't met anyone at school
- Other

**Question #21: Which accommodations have been suggested by the school?**

- My child got a tutor (teacher)
- Reduced timetable, personal support plan
- Homeschooling
- Change of class: repeat/skip grade, change of curriculum
- Change of school
- School exclusion
- The school has provided no accommodation
- Other

**Question #22: Did your child change schools because of school refusal?**

- Yes
- No

**Question #23: Has the school refusal led to judicial involvement or protective service report?**

- Yes
- No

**Question #24: Have you - or the other parent - adjusted your work schedule to adapt to the school refusal?**

- Yes
- No

**Question #25: Which health care follow-up has been provided to your child?**

- Psychiatrist /Child and Adolescent Psychiatrist
- Psychotherapist (Psychologist, Cognitive-Behavioral Therapy, Relaxation, Meditation, etc.)
- Speech therapy (i.e., for dyslexia)
- Social worker
- School counselor
- Other

**Question #26: How frequent were the appointments?**

- At least once a week
- At least once a month
- Less than once a month

**Question #27: Were the following assessments made?**

- Intelligence Test: IQ Test
- Brain imagery (i.e., MRI, scanner)
- Learning disorders screening (i.e., dyslexia, dysgraphia, dyscalculia, ADHD, etc.)
- Neurology appointment
- Autism Spectrum Disorder (ASD) screening
- No assessment has been made
- Other

**Question #28: Were other diagnoses made for your child?**

- No, no other diagnosis has been made
- High IQ > 130 ("gifted child")
- Learning disorder: dyslexia, dysgraphia, dyscalculia, dyspraxia
- ADHD
- Depression
- Bipolar disorder
- Autism Spectrum Disorder
- Schizophrenia
- Post-Traumatic Stress Disorder (PTSD)
- Social Phobia
- Generalized Anxiety Disorder (GAD)
- Obsessive Compulsive Disorder (OCD)
- Eating disorders
- Other

**Question #29: During school refusal, do you think that your child has had addictions**? *

* Addiction refers to dependence leading to compulsive behavior.

- Smartphone
- Video games
- Social media
- Smoking (cigarettes)
- Cannabis
- Alcohol
- Other substances (i.e., cocaine, ecstasy, LSD, etc.)

**Question #30: Did your child have one or several psychotherapies during school refusal?**

- Individual therapy
- Family therapy
- Group therapy (with peers)
- No, my child did not

**Question #31: Did your child receive medication for their school refusal?**

- Yes
- No

If yes, please select all medications that apply:

- Antidepressants (i.e., sertraline, fluoxetine, escitalopram, etc.)
- Anxiolytics (i.e., prazepam, bromazepam, diazepam, etc.)
- Antipsychotics (i.e., risperidone, aripiprazole, cyamemazine, etc.)
- Sleep pills (i.e., melatonin, zolpidem, prazosin, etc.)
- Herbal anxiolytics (i.e., valerian, passion flower, etc.)
- Other

Did you perceive this medication as efficient?

- Yes
- No

**Question #32: Was your child hospitalized because of school refusal?**

- Inpatient hospitalization (pediatrics, psychiatry)
- Intensive outpatient care (activities during the day at the hospital)
- No hospitalization
- Other

**Question #33: Did you or the other parent start psychotherapy or a psychiatry follow-up for yourself during your child's school refusal?**

- Yes
- No

**Question #34: Describe your relationship with your child's care providers after the onset of school refusal**

- Feeling of understanding: kindness and availability of at least some school workers
- Feeling of rejection: criticism, judgments, and threats
- Other

**Question #35 Did you have non-reimbursed healthcare expenses for your child's school refusal? (i.e., psychotherapy sessions, etc.)**

- Yes
- No

If yes: How much did you spend on these non-reimbursed healthcare expenses every month (in euros)? [numerical answer]

**Question #36: Did you have to renounce care for financial or organizational reasons?**

- Yes
- No
