# Supplementary material for: Trajectories of school refusal: sequence analysis using retrospective parent reports
Source: Eur Child Adolesc Psychiatry. 2024 Apr 11;33(11):3849–59. doi: 10.1007/s00787-024-02419-5 (PMC11588807; doi:10.1007/s00787-024-02419-5)
Supplement: Supplementary file 2 — Supplementary file2 (DOCX 31 KB) [file 787_2024_2419_MOESM2_ESM.docx]

**Appendix 2: Cluster Comparisons (Fisher Exact Test)**

We report all results with p < 0.01 and sort them out from the lowest p-value to the highest. Findings with high odd-ratios are highlighted in bold. Significance is displayed as *** for p ≤ 0.001 and ** for p < 0.01.

|  | Cluster Comparisons | p-value | Significance | Odd Ratio |
| --- | --- | --- | --- | --- |
| Complaints toward school | **{1,2,4} > {3,5}** | **< 0.001** | ******* | **1.83** |
|  | {1,2} > {3,4,5} | 0.001 | *** | 1.68 |
|  | {1} > {2,3,4,5} | 0.001 | ** | 1.65 |
|  | {1,4} > {2,3,5} | 0.001 | ** | 1.62 |
|  | {1,2,3,4} > {5} | 0.004 | ** | 1.75 |
| Complaints toward teachers | **{1,2,4} > {3,5}** | **< 0.001** | ******* | **2.48** |
|  | {1,2} > {3,4,5} | < 0.001 | *** | 1.95 |
|  | {1,2,3,4} > {5} | 0.001 | *** | 2.27 |
|  | {1,4} > {2,3,5} | 0.004 | ** | 1.63 |
|  | {1} > {2,3,4,5} | 0.009 | ** | 1.55 |
| Complaints toward classmates | **{1,2,4} > {3,5}** | **< 0.001** | ******* | **2.56** |
|  | {1,2} > {3,4,5} | < 0.001 | *** | 1.9 |
|  | {1,2,3,4} > {5} | < 0.001 | *** | 2.3 |
|  | {1,4} > {2,3,5} | 0.002 | ** | 1.66 |
|  | {1,2,4,5} > {3} | 0.007 | ** | 2.11 |
| Anger about going to school | **{4} > {1,2,3,5}** | **0.001** | ******* | **2.2** |
|  | {1,4} > {2,3,5} | 0.006 | ** | 1.56 |
| Opposition: Refusal to attend appointments or hostility toward authority figures | {1,4,5} > {2,3} | 0.001 | ** | 1.65 |
|  | **{1,2,4,5} > {3}** | **0.003** | ****** | **1.92** |
|  | **{4} > {1,2,3,5}** | **0.008** | ****** | **1.78** |
|  | {1,4} > {2,3,5} | 0.008 | ** | 1.44 |
| Headaches | **{1} > {2,3,4,5}** | **0.001** | ******* | **1.66** |
|  | {1,4} > {2,3,5} | 0.003 | ** | 1.55 |
| Stomachaches | **{1} > {2,3,4,5}** | **< 0.001** | ******* | **2.57** |
|  | {1,4} > {2,3,5} | < 0.001 | *** | 2.15 |
|  | {1,3} > {2,4,5} | 0.001 | ** | 1.93 |
|  | {1,3,4} > {2,5} | 0.002 | ** | 1.86 |
|  | {1,5} > {2,3,4} | 0.004 | ** | 1.75 |
|  | {1,4,5} > {2,3} | 0.005 | ** | 1.77 |
| Gender identity issues | **{2,3,5} > {1,4}** | **0.004** | ****** | **4.32** |
|  | {3,5} > {1,2,4} | 0.005 | ** | 3.53 |
| Aggravation of preexisting medical conditions (e.g., asthma, diabetes) | **{2} > {1,3,4,5}** | **< 0.001** | ******* | **2.86** |
|  | {2,4} > {1,3,5} | 0.003 | ** | 2.19 |
|  | {2,3} > {1,4,5} | 0.004 | ** | 2.13 |
|  | {1,2,3,4} > {5} | 0.006 | ** | 3.09 |
|  | {1,2} > {3,4,5} | 0.008 | ** | 2.02 |
|  | {1,2,4} > {3,5} | 0.009 | ** | 2.2 |
|  | {2,3,4} > {1,5} | 0.009 | ** | 1.92 |
| Learning disability (e.g., dyslexia, dyspraxia, dysgraphia, dyscalculia) | **{1,2} > {3,4,5}** | **0.001** | ******* | **1.78** |
|  | {1,2,3} > {4,5} | 0.002 | ** | 1.81 |
| Social phobia | {3,4,5} > {1,2} | < 0.001 | *** | 1.87 |
|  | {3,5} > {1,2,4} | < 0.001 | *** | 1.83 |
|  | {4,5} > {1,2,3} | 0.001 | *** | 1.75 |
|  | **{5} > {1,2,3,4}** | **0.001** | ******* | **1.88** |
|  | {2,3,4,5} > {1} | 0.001 | ** | 1.69 |
|  | {2,3,5} > {1,4} | 0.007 | ** | 1.49 |
| SR diagnosis by a health care provider | {3,5} > {1,2,4} | < 0.001 | *** | 2.33 |
|  | {3,4,5} > {1,2} | < 0.001 | *** | 2.07 |
|  | **{3} > {1,2,4,5}** | **0.001** | ****** | **2.54** |
|  | {2,3,4,5} > {1} | 0.002 | ** | 1.65 |
|  | {2,3,5} > {1,4} | 0.004 | ** | 1.59 |
|  | {5} > {1,2,3,4} | 0.008 | ** | 1.79 |
|  | {3,4} > {1,2,5} | 0.009 | ** | 1.68 |
| Follow up by a psychiatrist / child and adolescent psychiatrist | **{3,4,5} > {1,2}** | **< 0.001** | ******* | **2.16** |
|  | {4,5} > {1,2,3} | < 0.001 | *** | 1.94 |
|  | {3,5} > {1,2,4} | < 0.001 | *** | 1.88 |
|  | {3,4} > {1,2,5} | 0.002 | ** | 1.78 |
|  | {2,3,4,5} > {1} | 0.002 | ** | 1.62 |
|  | {5} > {1,2,3,4} | 0.006 | ** | 1.76 |
| Frequency of care appointement: at least once a week | NS | | | |
| Psychotherapy >1 type: individual; family, group therapy | **{5} > {1,2,3,4}** | **< 0.001** | ******* | **2.15** |
|  | {4,5} > {1,2,3} | < 0.001 | *** | 1.84 |
|  | {2,4,5} > {1,3} | < 0.001 | *** | 1.74 |
|  | {2,3,4,5} > {1} | < 0.001 | *** | 1.79 |
|  | {2,5} > {1,3,4} | 0.001 | *** | 1.72 |
|  | {3,4,5} > {1,2} | 0.001 | *** | 1.69 |
|  | {3,5} > {1,2,4} | 0.001 | *** | 1.72 |
|  | {2,3,5} > {1,4} | 0.001 | ** | 1.64 |
| Inpatient hospitalization (in psychiatry or pediatrics) | **{5} > {1,2,3,4}** | **0.001** | ******* | **1.95** |
|  | {3,4,5} > {1,2} | 0.001 | *** | 1.71 |
|  | {4,5} > {1,2,3} | 0.001 | ** | 1.73 |
|  | {3,5} > {1,2,4} | 0.001 | ** | 1.72 |
|  | {2,3,4,5} > {1} | 0.009 | ** | 1.55 |
| Antidepressant | **{3,4,5} > {1,2}** | **< 0.001** | ******* | **2.06** |
|  | {3,4} > {1,2,5} | < 0.001 | *** | 1.94 |
|  | {3,5} > {1,2,4} | < 0.001 | *** | 1.8 |
|  | {2,3,4,5} > {1} | 0.001 | ** | 1.64 |
|  | {3} > {1,2,4,5} | 0.001 | ** | 1.99 |
|  | {4,5} > {1,2,3} | 0.004 | ** | 1.56 |
| Antipsychotic | **{5} > {1,2,3,4}** | **0.002** | ****** | **3.0** |
|  | {4,5} > {1,2,3} | 0.003 | ** | 2.66 |
| Medication perceived as efficient | **{3} > {1,2,4,5}** | **0.005** | ****** | **2.11** |
|  | {1,3} > {2,4,5} | 0.01 | ** | 1.65 |
| >1 type of medication: antidepressant; anxiolytic; sleeping pill; ADHD medication; antipsychotic; herbal anxiolytic | **{3,4,5} > {1,2}** | **< 0.001** | ******* | **1.8** |
|  | {3,4} > {1,2,5} | 0.001 | *** | 1.79 |
|  | **{1,3,4,5} > {2}** | **0.004** | ****** | **1.89** |
| Judicial involvement or protective service report | **{4,5} > {1,2,3}** | **0.001** | ******* | **2.1** |
|  | {2,4,5} > {1,3} | 0.001 | *** | 2.06 |
|  | {5} > {1,2,3,4} | 0.004 | ** | 1.98 |
| Separation of children from parents suggested | **{3,4,5} > {1,2}** | **< 0.001** | ******* | **2.19** |
|  | {3,5} > {1,2,4} | < 0.001 | *** | 2.12 |
|  | **{5} > {1,2,3,4}** | **< 0.001** | ******* | **2.28** |
|  | {4,5} > {1,2,3} | < 0.001 | *** | 2.06 |
|  | {2,3,4,5} > {1} | 0.004 | ** | 1.66 |
|  | {1,3,4,5} > {2} | 0.009 | ** | 1.82 |
| School accommodation (reduced timetable, personal support plan) | **{2,3,4} > {1,5}** | **0.001** | ******* | **1.66** |
|  | **{2,3,4,5} > {1}** | **0.001** | ******* | **1.67** |
|  | {3,4} > {1,2,5} | 0.005 | ** | 1.59 |
|  | {2,3} > {1,4,5} | 0.005 | ** | 1.54 |
| Change of school | **{2,3,4,5} > {1}** | **0.001** | ******* | **1.66** |
|  | {3,4,5} > {1,2} | 0.001 | ** | 1.6 |
|  | {3,5} > {1,2,4} | 0.003 | ** | 1.56 |
|  | {2,3,5} > {1,4} | 0.005 | ** | 1.48 |
| Home schooling | **{4,5} > {1,2,3}** | **< 0.001** | ******* | **2.75** |
|  | {3,4,5} > {1,2} | < 0.001 | *** | 2.31 |
|  | {2,4,5} > {1,3} | < 0.001 | *** | 2.3 |
|  | {2,3,4,5} > {1} | < 0.001 | *** | 2.27 |
|  | **{5} > {1,2,3,4}** | **< 0.001** | ******* | **2.4** |
|  | {3,5} > {1,2,4} | 0.001 | ** | 1.75 |
|  | {2,5} > {1,3,4} | 0.002 | ** | 1.68 |
|  | {4} > {1,2,3,5} | 0.005 | ** | 1.93 |
| Meeting with the education officer | **{1,3,4,5} > {2}** | **0.003** | ****** | **1.72** |
| Relationship with school: sense of rejection | **{2} > {1,3,4,5}** | **0.005** | ****** | **1.71** |
