# Supplementary material for: Trajectories of school refusal: sequence analysis using retrospective parent reports
Source: Eur Child Adolesc Psychiatry. 2024 Apr 11;33(11):3849–59. doi: 10.1007/s00787-024-02419-5 (PMC11588807; doi:10.1007/s00787-024-02419-5)
Supplement: Supplementary file 3 — Supplementary file3 (DOCX 729 KB) [file 787_2024_2419_MOESM3_ESM.docx]

**Supplementary Figure 1: Dendrogram of School Refusal trajectories**
**
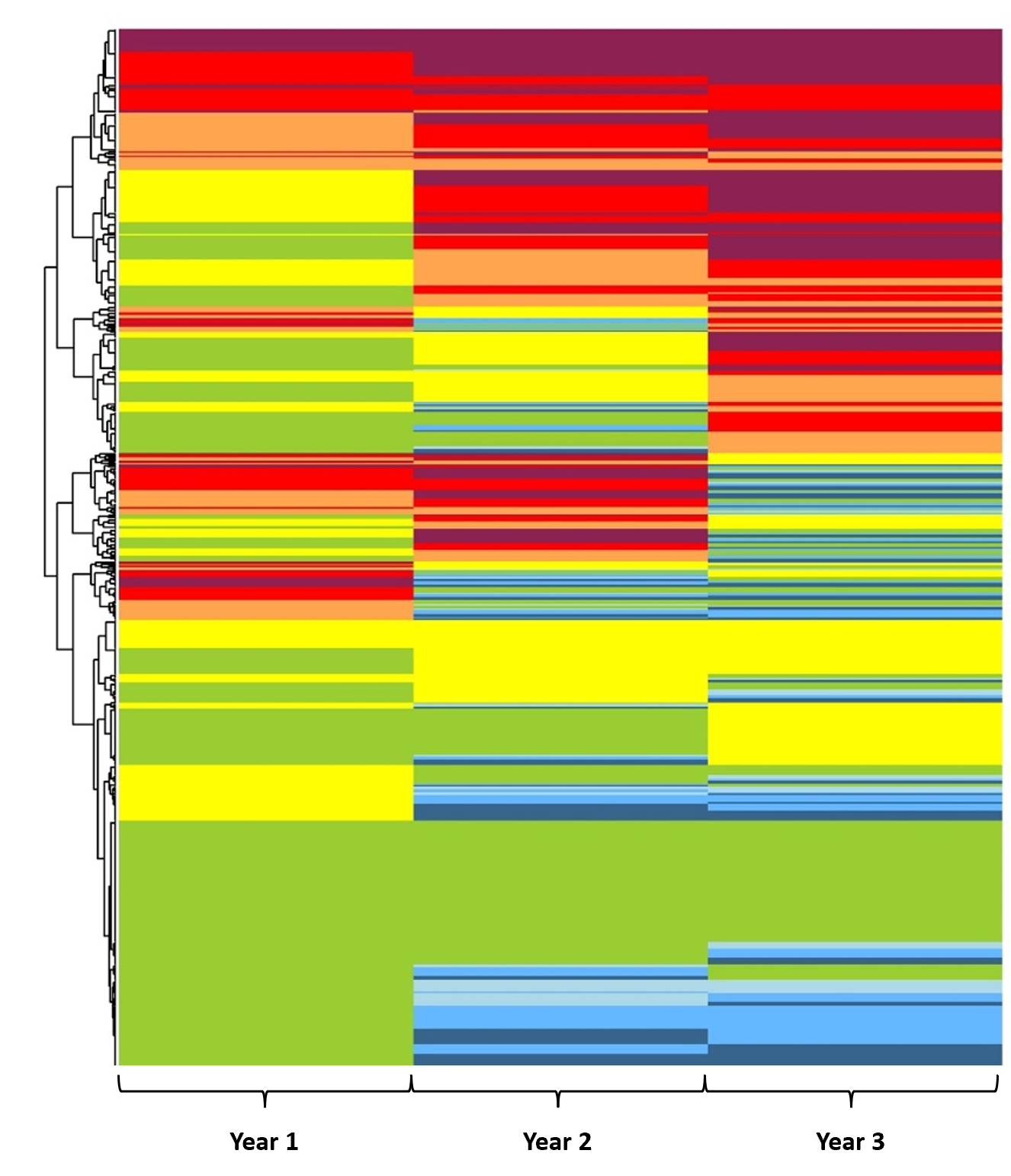
**

The analysis of back-to-school trajectories was analyzed in sequences, which were represented in the form of a dendrogram. A line from the dendrogram depicted a child. Each of the three columns represents one year. Blue is associated with a low absence from school and red with a high absence from school. We can see that some children never go back to school during the three years of follow-up. Others go to school between 1 month and 1 term and then never go back. Some children do not go to school and then go back completely. Others still have absences of between 2 and 1 month during the 3 years of follow-up. And finally, some children have returned to school completely after an absence of less than a month. These sequences, once grouped into clusters, develop the identification of the different back-to-school profiles.

Color code:


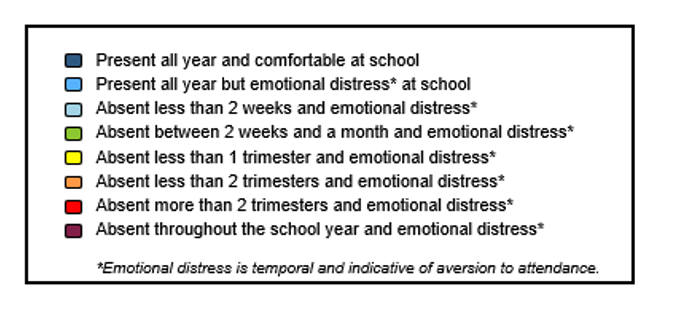


**Supplementary Figure 2: Dissimilarities between sequences**


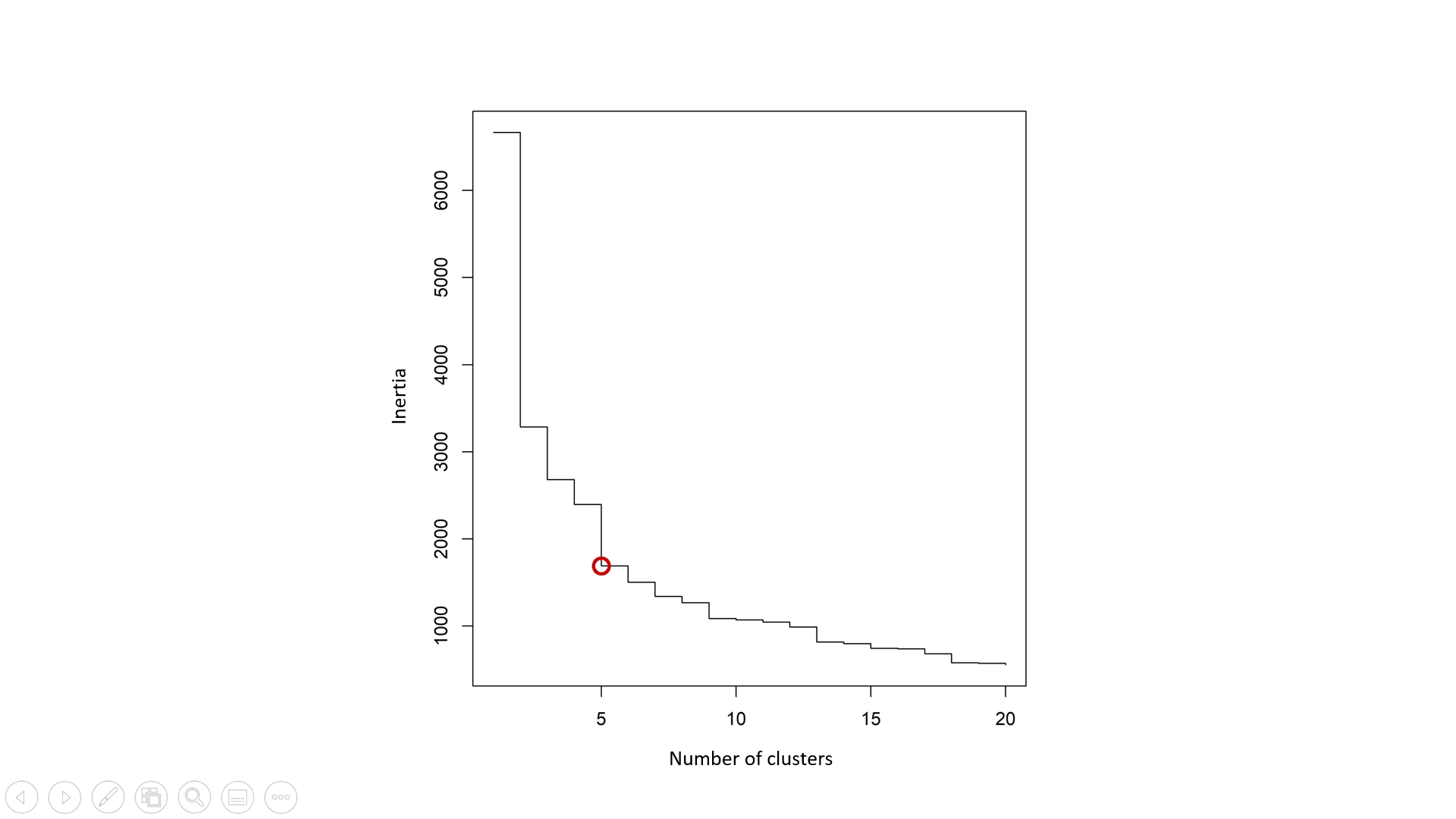


We tested different methods for calculating the number of clusters such as the inertia block (the largest drop in inertia gives the group number) with the bestcutree function of the R JLutils library (version 1.16.0). Following a visual inspection of the clusters obtained during these tests and the cluster partitioning indices, we were able to confirm that the optimal number of clusters is 5.
